# Alcohol and breast cancer risk: the alcoholism paradox

**DOI:** 10.1054/bjoc.2000.1360

**Published:** 2000-09-04

**Authors:** H Kuper, W Ye, E Weiderpass, A Ekbom, D Trichopoulos, O Nyrén, H-O Adami

**Affiliations:** Department of Epidemiology and Harvard Center for Cancer Prevention, Harvard University, Boston, MA 02115, USA; Department of Medical Epidemiology, Karolinska Institutet, PO Box 281, Stockholm, 171 77, Sweden

**Keywords:** alcoholism, alcohol, breast cancer

## Abstract

A population-based cohort study of 36 856 women diagnosed with alcoholism in Sweden between 1965 and 1995 found that alcoholic women had only a small 15% increase in breast-cancer incidence compared to the general female population. It is therefore apparent, contrary to expectation, that alcoholism does not increase breast-cancer risk in proportion to presumed ethanol intake. © 2000 Cancer Research Campaign


British Journal of Cancer (2000) 83(7), 949–951
© 2000 Cancer Research Campaign
doi: 10.1054/ bjoc.2000.1360, available online at http://www.idealibrary.com on IDEAL®

### Alcohol and breast cancer risk: the alcoholism paradox

**H Kuper[1], W Ye[2], E Weiderpass[2], A Ekbom[1,2], D Trichopoulos[1,2], O Nyrén[2] and H-O Adami[1,2]**

[1]Department of Epidemiology and Harvard Center for Cancer Prevention, Harvard University, Boston, MA 02115, USA; [2]Department of Medical Epidemiology, Karolinska Institutet, PO Box 281, 171 77 Stockholm, Sweden

**Summary** A population-based cohort study of 36 856 women diagnosed with alcoholism in Sweden between 1965 and 1995 found that alcoholic women had only a small 15% increase in breast-cancer incidence compared to the general female population. It is therefore apparent, contrary to expectation, that alcoholism does not increase breast-cancer risk in proportion to presumed ethanol intake. © 2000 Cancer Research Campaign

**Keywords**: alcoholism; alcohol; breast cancer

Breast cancer is a major public health problem in Western countries, not withstanding the progress made in the treatment of the disease (Cole and Rodu, 1996). Prevention of disease would be preferable, but of the more than 20 risk factors identified for the disease (Adami et al, 1998) only one is modifiable and associated with a substantial risk gradient: alcohol consumption (50 g of ethanol or five drinks per day) increases the risk by about 40% (Smith-Warner et al, 1998). Because there is an exposure response trend for most factors that have been shown to increase the risk of cancer, it would be reasonable to assume that very heavy alcohol consumption (> 60 g of ethanol or more than six drinks per day) would further increase the risk of breast cancer. Somewhat surprisingly, several studies (Hiatt and Bawol, 1984; Lê et al, 1984; Longnecker et al; 1995*a*; 1995*b*; Thun et al, 1997), as well as a pooled analysis of cohort studies (Smith-Warner et al, 1998), showed some evidence of a plateau of breast-cancer risk among women consuming very large quantities of alcohol. Limited statistical power and concern about misclassification of alcohol intake, however, hampered further interpretation (Smith-Warner et al, 1998). In order to adequately investigate how extreme levels of alcohol intake affect breast-cancer risk, we have undertaken a population-based cohort study in Sweden, utilizing nationwide registry data. The present study is the largest study of breast-cancer risk among female alcoholics ever to be carried out (Prior, 1988; Adami et al, 1992; Tonnesen et al, 1994; Sigvardsson et al, 1996).

### MATERIALS AND METHODS

In Sweden there is virtually no private inpatient treatment. Hospital-provided medical services are population-based and referable to the county in which the patient lives. From 1965 onwards, the National Board of Health and Welfare started collecting data on individual hospital discharges in the Inpatient Register. From 1987, the register attained complete nationwide coverage. All patients recorded in the Inpatient Register with a discharge diagnosis of alcoholism were initially selected for inclusion in the study. A total of 196 803 individually unique national registration numbers, assigned to all Swedish residents, were registered at least once with a diagnosis of alcoholism between 1965 and 1994. December 31, 1995 was the end of the observation period.

Record linkage of the study cohort to the nationwide Registers of Causes of Death, Emigration and Cancer allowed the calculation of follow-up time, in person-years, of eligible persons at risk as described previously in detail (Adami et al, 1992; 1996). From the total cohort 7790 records were excluded because of erroneous or incomplete national registration numbers, a further 3405 patients were excluded because they had prevalent cancers at the time observation began and another 2941 patients because of inconsistencies uncovered during record linkage. Thus a total of 182 667 patients with alcoholism remained eligible, and of these 36 856 were women.

The expected number of cancers was calculated by multiplying the number of observed person-years by age (in 5-year groups), sex, and calendar year-specific cancer incidence rates of first breast cancers. These rates, derived from the entire Swedish population and aggregated by 5 calendar years to avoid instability, were calculated by dividing the number of first breast cancers, excluding those discovered incidentally at autopsy, by person-years at risk (number of mid-year population without a previously reported cancer diagnosis). The main outcome, the standardized incidence ratio (SIR), was calculated as the ratio of the observed number of cancers to that expected. The 95% confidence interval (CI) of the SIR was calculated on the basis of the Poisson distribution. In main analyses, we excluded cancers and person-years accumulated during the first year of follow-up in order to minimize the possible impact of selection and detection biases (Berkson, 1946).

As the studied potential determinants of breast-cancer risk, including certain diseases among the alcoholic women, may covary and thus confound each other, we modelled breast-cancer risk in relation to age at follow-up, duration of follow-up, calendar year at enrolment to the cohort, and presence of diabetes or liver cirrhosis, using Poisson regression by taking the expected number of cases in the offset.

*Received 8 May 2000*
*Revised 26 May 2000*
*Accepted 31 May 2000*

*Correspondence to*: H-O Adami[2]

**Table 1** Breast cancer incidence between 1966 and 1995 among 36 856 women who were hospitalized for alcoholism at least 1 year prior to the diagnosis of breast cancer

| Determinant | Observed cases | Standardized incidence ratio | 95% confidence interval |
|---|---|---|---|
| Years of follow-up | | | |
| 1–30* | 514 | 1.15 | 1.05–1.25 |
| < 1 | 40 | 1.09 | 0.78–1.49 |
| 1–9 | 331 | 1.21 | 1.08–1.35 |
| ≥ 10 | 183 | 1.06 | 0.91–1.22 |
| Age at follow-up* (years) | | | |
| <50 | 143 | 1.11 | 0.93–1.30 |
| 50–59 | 153 | 1.16 | 0.98–1.36 |
| 60–69 | 129 | 1.14 | 0.95–1.35 |
| ≥ 70 | 89 | 1.24 | 0.99–1.52 |

*Excluding the first year of follow-up

**Table 2** Poisson regression-derived relative risk (RR) with 95% confidence interval (CI) of risk for breast cancer*

| Determinant | RR | 95% CI |
|---|---|---|
| Age at follow-up (years) | | |
| < 50 | 1.00 | Reference |
| 50–59 | 1.06 | 0.84–1.33 |
| 60–69 | 1.07 | 0.91–1.53 |
| ≥ 70 | 1.18 | 0.91–1.53 |
| Duration of follow-up (years) | | |
| 1–9 | 1.00 | Reference |
| ≥ 10 | 0.84 | 0.69–1.02 |
| Calendar year at enrolment | | |
| 1965–1974 | 1.00 | Reference |
| 1975–1984 | 0.85 | 0.69–1.06 |
| 1985–1994 | 0.87 | 0.66–1.15 |
| Diabetes mellitus | | |
| No | 1.00 | Reference |
| Yes | 0.88 | 0.62–1.25 |
| Liver cirrhosis | | |
| No | 1.00 | Reference |
| Yes | 0.93 | 0.65–1.33 |

*Cancers and person-years accrued during the first year of follow-up were excluded. The baseline standardized incidence ratio for patients with all the reference characteristics was estimated to be 1.31

### RESULTS

The mean age at entry for the 36 856 women with a diagnosis of alcoholism in the study was 42.7 years, and the mean duration of follow-up was 9.6 years, yielding a total of 353 596 woman-years at risk. Among these women 2352 (6.4%) also had a diagnosis of liver cirrhosis in at least one hospitalization before the diagnosis of breast cancer; the corresponding figure for diabetes mellitus was 2003 (5.4%). The mean calendar year at enrolment was 1983.

The results based on external comparisons are summarized in Table 1. We found a statistically significant overall excess risk of breast cancer among women with a previous diagnosis of alcoholism. The excess, however, is small, amounting to about 15% over the age-adjusted average risk among Swedish women. We found no evidence for an important variation in risk over 1–30 years of follow-up, nor any difference with age at diagnosis of breast cancer or with menopausal status, using age 50 to crudely separate the pre- and post-menopausal women.

Results based on internal comparisons within the cohort of alcoholics and generated by Poisson regression are shown in Table 2. Notwithstanding mutual adjustment, we found no convincing trends with age at follow-up, duration of follow-up or calendar year of entry, the latter used as a marker of changes in diagnostic practices. Neither diabetes nor liver cirrhosis appeared to be significantly related to risk and any associations may be due to chance.

### DISCUSSION

Alcoholic women are characterized by elevated serum levels of oestradiol (Gavaler and van Thiel, 1992). Post-menopausal alcoholic women with cirrhosis have, in addition, low levels of testosterone, follicle stimulating hormone and luteinizing hormone (Gavaler and van Thiel, 1992). Moreover, among alcoholic women there appears to be a derangement of the usual pattern of association among the various hormones, indicating a disruption of the normal feedback mechanisms (Gavaler and van Thiel, 1992). Both alcohol intake and elevation of oestrogen levels would argue for a substantial increase in breast-cancer risk, but instead the increase in risk observed in our study was fairly small.

Because our study used a cohort design and a nationwide source population with virtually complete follow-up, we consider selection and information bias unlikely, especially since the first year of follow-up was excluded. It is possible, but not well substantiated, that women who are alcoholics compared to the background population have an earlier age at first full-term pregnancy, more pregnancies, and – as a consequence of their higher smoking prevalence – an earlier menopause, as well as being generally of lower socioeconomic status. These risk factors are, however, too weakly associated with breast-cancer risk in this Swedish population to introduce more than minimal underestimation of risk (Adami et al, 1998). Concerns about confounding by body mass index – which has a known dual effect on breast-cancer risk (Adami et al, 1998) – are offset by the similar risks among pre- and post-menopausal women, and the potential confounding effect of diabetes mellitus was controlled for in the Poisson analysis (Weiderpass et al, 1997). Lastly, associations with use of oral contraceptives and hormone replacement therapy in relation to breast-cancer risk are weak (Collaborative Group on Hormonal Factors in Breast Cancer, 1997; 1998) and low use among alcoholic women has not been demonstrated. A recent study in a partially overlapping population (Magnusson et al, 1999) and in other settings (Hiatt and Bawol, 1984; Lê et al, 1984; Longnecker et al, 1995*a*) with detailed adjustment for confounding provides further reassurance that confounding is of limited concern in studies of alcohol and breast cancer.

As the Swedish Inpatient Register did not contain any indices of disease severity, we were unable to study dose-response. Attempts to use number of hospitalizations per unit of time (hospitalization density) as a proxy for severity had to be given up because of survival bias; a low hospitalization density presupposed a long follow-up time, and since censoring occurred at the time of cancer diagnosis, a long follow-up time followed by a new admission for alcoholism (and not cancer) was, in practice, without risk for cancer.

There are several possible explanations for our finding that the risk for breast cancer does not further increase with very high, as contrasted to high (~50 g of ethanol per day), alcohol intake. First, amenorrhoea (Mello et al, 1996) and a breakdown of normal hormonal feedback mechanisms (Gavaler and van Thiel, 1992) have been reported to be relatively frequent among alcoholic

*© 2000 Cancer Research Campaign*

women, particularly among those with cirrhosis, thus reducing breast-cancer risk. Secondly, experimental and human data indicate that high levels of oestrogens may down-regulate oestrogen receptor-α expression (Lawson et al, 1999) that, in turn, limits expression of oestrogenic effects including breast-cancer progression. Last, in studies in female rodents, Hilakivi-Clarke (1996) has noticed that a deficit of oestradiol exposure tends to increase alcohol consumption. Nevertheless, our data challenge a monotonic causal association between alcohol and breast cancer.

In conclusion, this study indicates that, contrary to expectation, alcoholism does not increase breast-cancer risk in proportion to presumed ethanol intake. Nevertheless, our findings do not challenge the existence of a positive association between alcohol intake and breast-cancer risk, nor do they contradict the exposure–response pattern reported by other investigators at lower levels of alcohol intake.

### ACKNOWLEDGEMENTS

This study was supported by several grants from the Swedish Cancer Society.

### REFERENCES

Adami HO, McLaughlin JK, Hsing AW, Wolk A, Ekbom A, Holmberg L and Persson I (1992) Alcoholism and cancer risk: a population-based cohort study. *Cancer Causes Control* **3**: 419–425

Adami HO, Chow WH, Nyren O, Berne C, Linet MS, Ekbom A, Wolk A, McLaughlin JK and Fraumeni JF Jr (1996) Excess risk of primary liver cancer in patients with diabetes mellitus. *J Natl Cancer Inst* **88**: 1472–1477

Adami HO, Signorello LB and Trichopoulos D (1998) Towards an understanding of breast cancer etiology. *Semin Cancer Biol* **8**: 255–262

Berkson J (1946) Limitations of the application of fourfold table analysis to hospital data. *Biomet Bull* **2**: 47–53

Cole P and Rodu B (1996) Declining cancer mortality in the United States. *Cancer* **78**: 2045–2048

Collaborative Group on Hormonal Factors in Breast Cancer (1997) Breast cancer and hormone replacement therapy: collaborative re-analysis of data from 51 epidemiological studies of 52,705 women with breast cancer and 108,411 women without breast cancer. *Lancet* **350**: 1047–1059

Collaborative Group on Hormonal Factors in Breast Cancer (1998) Breast cancer and hormonal contraceptives: collaborative reanalysis of individual data on 53 297 women with breast cancer and 100 239 women without breast cancer from 54 epidemiological studies. *Lancet* **347**: 1713–1727

Gavaler JS and van Thiel DH (1992) Hormonal status of postmenopausal women with alcohol-induced cirrhosis: further findings and a review of the literature. *Hepatology* **16**: 312–319

Hiatt RA and Bawol RD (1984) Alcoholic beverage consumption and breast cancer incidence. *Am J Epidemiol* **120**: 676–683

Hilakivi-Clarke L (1996) Role of estradiol in alcohol intake and alcohol-related behaviors. *J Stud Alcohol* **57**: 162–170

Lawson JS, Field AS, Champion S, Tran D, Ishikura H and Trichopoulos D (1999) Low oestrogen receptor alpha expression in normal breast tissue underlies low breast cancer incidence in Japan. *Lancet* **354**: 1787–1788

Lê MG, Hill C, Kramar A and Flamanti R (1984) Alcoholic beverage consumption and breast cancer in a French case-control study. *Am J Epidemiol* **120**: 350–357

Longnecker MP, Newcomb PA, Mittendorf R, Greenberg ER, Clapp RW, Bogdan GF, Baron J, MacMahon B and Willett WC (1995*a*) Risk of breast cancer in relation to lifetime alcohol consumption. *J Natl Cancer Inst* **87**: 923–929

Longnecker MP, Paganini-Hill A and Ross RK (1995*b*) Lifetime alcohol consumption and breast cancer risk among postmenopausal women in Los Angeles. *Cancer Epidemiol Biomarkers Prev* **4**: 721–725

Magnusson C, Baron JA, Correia N, Bergstrom R, Adami HO and Persson I (1999) Breast-cancer risk following long-term oestrogen- and oestrogen-progestin-replacement therapy. *Int J Cancer* **81**: 339–344

Mello NK, Mendelson JH and Teoh SK (1989) Neuroendocrine consequences of alcohol abuse in women. *Ann N Y Acad Sci* **562**: 211–240

Prior P (1988) Long-term cancer risk in alcoholism. *Alcohol Alcohol* **23**: 163–171

Sigvardsson S, Hardell L, Przybeck TR and Cloninger R (1996) Increased cancer risk among Swedish female alcoholics. *Epidemiology* **7**: 140–143

Smith-Warner SA, Spiegelman D, Yaun SS, van den Brandt PA, Folsom AR, Goldbohm RA, Graham S, Holmberg L, Howe GR, Marshall JR, Miller AB, Potter JD, Speizer FE, Willett WC, Wolk A and Hunter DJ (1998) Alcohol and breast cancer in women: a pooled analysis of cohort studies. *JAMA* **279**: 535–540

Thun MJ, Peto R, Lopez AD, Monaco JH, Henley SJ, Heath CW Jr and Doll R (1997) Alcohol consumption and mortality among middle-aged and elderly U.S. adults. *N Engl J Med* **337**: 1705–1714

Tonnesen H, Moller H, Andersen JR, Jensen E and Juel K (1994) Cancer morbidity in alcohol abusers. *Br J Cancer* **69**: 327–332

Weiderpass E, Gridley G, Persson I, Nyren O, Ekbom A and Adami HO (1997) Risk of endometrial and breast cancer in patients with diabetes mellitus. *Int J Cancer* **71**: 360–363

*© 2000 Cancer Research Campaign*
